# Identification of Brain Damage after Seizures Using an MR-Based Electrical Conductivity Imaging Method

**DOI:** 10.3390/diagnostics11030569

**Published:** 2021-03-22

**Authors:** Sanga Kim, Bup Kyung Choi, Ji Ae Park, Hyung Joong Kim, Tong In Oh, Won Sub Kang, Jong Woo Kim, Hae Jeong Park

**Affiliations:** 1 Department of Pharmacology, School of Medicine, Kyung Hee University, Seoul 02447, Korea; sanga0568@naver.com; 2 Department of Biomedical Engineering, School of Medicine, Kyung Hee University, Seoul 02447, Korea; josh_bk@naver.com (B.K.C.); bmekim@khu.ac.kr (H.J.K.); 3 Division of Applied RI, Korea Institute of Radiological & Medical Science, Seoul 01812, Korea; jpark@kirams.re.kr; 4 Department of Neuropsychiatry, School of Medicine, Kyung Hee University, Seoul 02447, Korea; menuhinwskang@khu.ac.kr

**Keywords:** seizure, N-methyl-D-aspartate, neuronal cell death, magnetic resonance electrical property tomography, hippocampus

## Abstract

Previous imaging studies have shown the morphological malformation and the alterations of ionic mobility, water contents, electrical properties, or metabolites in seizure brains. Magnetic resonance electrical properties tomography (MREPT) is a recently developed technique for the measurement of electrical tissue properties with a high frequency that provides cellular information regardless of the cell membrane. In this study, we examined the possibility of MREPT as an applicable technique to detect seizure-induced functional changes in the brain of rats. Ultra-high field (9.4 T) magnetic resonance imaging (MRI) was performed, 2 h, 2 days, and 1 week after the injection of N-methyl-D-aspartate (NMDA; 75 mg/kg). The conductivity images were reconstructed from B1 phase images using a magnetic resonance conductivity imaging (MRCI) toolbox. The high-frequency conductivity was significantly decreased in the hippocampus among various brain regions of NMDA-treated rats. Nissl staining showed shrunken cell bodies and condensed cytoplasm potently at 2 h after NMDA treatment, and neuronal cell loss at all time points in the hippocampus. These results suggest that the reduced electrical conductivity may be associated with seizure-induced neuronal loss in the hippocampus. Magnetic resonance (MR)-based electrical conductivity imaging may be an applicable technique to non-invasively identify brain damage after a seizure.

## 1. Introduction

Epilepsy is a neurological disorder characterized by recurrent and long-lasting seizures. A seizure is a paroxysmal symptom induced by abnormally excessive excitatory activity in the brain [1]. Previous studies have reported that seizures occur due to the excessive release of glutamate [2,3]. A microdialysis study for epilepsy patients showed an increased concentration of glutamate in the hippocampus during seizures [4,5]. In addition, rodents treated with glutamate agonists, such as N-methyl-D-aspartate (NMDA) and kainic acid (KA), have been used as seizure animal models, showing tonic–clonic seizures [6,7]. The excessive release of glutamate promotes an excessive activation of glutamate receptors and an intracellular Ca^2+^ overload, which triggers excitotoxic cell death [8]. Indeed, previous studies have shown that the glutamate agonists induced neuronal cell death in the rodent brains by regulating the cell death-related proteins, such as caspase 3 (CASP3), BCL2, and P38, along with seizures [9,10,11].

Previous studies have shown the morphological malformation and the alterations of ionic mobility, water content, or metabolites in the brains of patients with seizures. In patients with seizures, magnetic resonance imaging (MRI) showed unilateral or bilateral lesions at the hippocampus, cortex, and thalamic pulvinar nucleus through high T2 signals [12]. Positron emission tomography (PET) showed the negative correlation of the glucose metabolism with the duration of the spontaneous recurrent seizures, suggesting the structural and functional abnormalities of the brain during epileptogenesis [13]. Diffusion-weighted imaging (DWI) through measuring the afferent diffusion coefficient (ADC) revealed that the water influx into the cells was increased to sustain electrolyte homeostasis in patients’ brains after seizures [14]. Alteration of the electrical activity in seizure brains has been also reported. Electroencephalograms (EEGs), which are widely taken for the diagnosis of seizure, show abnormal patterns, such as focal spikes or sharp waves, defined as epileptic activity [15]. An electrical impedance tomography (EIT) study reported the optimal frequency for epileptiform activity (1355 Hz) in the brain of epileptic rats [16].

Magnetic resonance electrical properties tomography (MREPT) is a recently proposed, non-invasive imaging technique to measure electrical tissue properties using an MRI scanner. Electrical properties, like conductivity and permittivity, represent the ionic concentration and mobility across the cell membrane [17]. MREPT yields the quantitative values for the high-frequency conductivity, determined at the Larmor frequency of MRI, and provides novel contrast compared with the ordinarily used T2-weighted or DWI–ADC image techniques [17]. In particular, MREPT has advantages, including non-invasiveness, high spatial resolution, and superior feasibility for in vivo studies without injecting an imaging current, compared to other electrical property (EP) techniques. Indeed, MREPT has shown different conductivities between injured and normal brains in canines [18]. Not only was the increased conductivity determined in the central lesions of the abscessed brain [18], but also the time-dependent decrease of the conductivity by the destruction of tissues during euthanasia was detected in the normal canine brain [19].

Considering these previous reports, we expected that the high-frequency conductivity could represent the pathophysiological changes of a seizure-induced damaged brain. In NMDA-treated seizure rats, we reconstructed the conductivity images of the brains at various time points (2 h, 2 days, and 1 week) after seizure using an ultra-high-field (9.4 T) MRI scanner with a spatial resolution of 0.3 × 0.3 × 1 mm^3^. In addition, we examined the changes in the morphology and biological molecules, such as neuronal nuclei (NeuN), CASP3, and glial fibrillary acidic protein (GFAP) in the brain of NMDA-treated rats.

## 2. Materials and Methods

### 2.1. Animals and N-Methyl-D-Aspartic Acid (NMDA) Treatment

A total of forty male Sprague–Dawley rats (7 weeks old, 250–270 g) were provided by Orient Bio (Gyeonggi-do, Korea). Rats were housed in controlled temperature and humidity (25 ± 2 °C, 50 ± 5%) under a 12 h light–dark cycle with free access to food and water. Animal experiments were approved by IACUC of Kyung Hee University (KHUASP-18-137), and were performed in accordance with the animal care guidelines of the Korean Academy of Medical Sciences.

The rats were randomly divided into control (*n* = 10) and NMDA treatment groups (*n* = 10 per group (2 h, 2 days, and 1 week after NMDA treatment groups)). NMDA (75 mg/kg; AK Scientific, Union City, CA, United States) was injected intraperitoneally to rats of NMDA treatment groups on the same day. The rats were then sacrificed at 2 h (*n* = 10), 2 days (*n* = 10), and 1 week (*n* = 10) after the injection, sequentially. Rats of the control group were intraperitoneally treated with saline, and were maintained with the same schedule as the 1 week NMDA treatment group. All of the rats completed the entire protocol without missing any (due to death during experimental procedures or humane endpoints).

### 2.2. Assessment of Seizure Severity

The seizure behaviors of the rats were recorded in each cage for 1 h after NMDA injection. The severity of seizures was assessed using a modified Racine’s scale [20,21]. Seizures were scored as follows: 0 = normal; 1 = motionless; 2 = tail stiffness; 3 = lordotic posture and whip-like tail; 4 = forelimb clonus with rearing and falling, body twist; and 5 = generalized tonic–clonic activity.

### 2.3. Conductivity Image of Ultra-High-Field (9.4 T) MRI

We measured the electrical properties in the brain at 2 h, 2 days, and 1 week after NMDA treatment using an ultra-high-field (9.4 T) MRI scanner (Agilent Technologies, Santa Clara, CA, USA) with a single-channel body coil. The rats were anesthetized with 3% isoflurane during the MRI scan. For the high-frequency conductivity image reconstruction, the B1 phase maps were acquired using the multi-echo spin-echo imaging sequence with a pixel size of 0.3 × 0.3 mm^2^. The imaging parameters were as follows: TR/TE (time to repetition /time to echo) = 2200/22, 44, 66, 88, 110, 132 ms; number of signal acquisitions = 5; field-of-view (FOV) = 40 × 40 mm^2^; slice thickness = 1 mm; flip angle = 90°; and image matrix size = 128 × 128.

Six echoes were used during the total scan time (23 min). For the conductivity image reconstruction, the acquired B1 phase map was unwrapped using the PUMA (Phase Unwrapping via MAx flows) algorithm [22]. The multi-echo signal was then averaged using a weight factor to improve the signal-to-noise ratio (SNR) [17]. The optimized B1 phase map was then used to reconstruct the high-frequency conductivity using an in-house-developed, magnetic resonance conductivity imaging (MRCI) toolbox [23]. For the quantitative analysis of brain regional conductivity, ROIs (regions of interest) were determined with reference to the results of previous studies [12,13,14], including the amygdala, medial thalamus, hippocampus, temporal cortex, frontal cortex, parietal cortex, cingulate cortex, caudate putamen. Since the MRCI toolbox generates conductivity distribution, which was co-registered with anatomical magnetic resonance (MR) images, we assigned the ROIs on the MR images by means of geometric relation with respect to prominent anatomical landmarks, using the rat brain atlas [24], and then segmented the ROIs from the corresponding conductivity images. The ROI was also confirmed by an experienced radiologist, and finally conductivity value was calculated.

### 2.4. Tissue Collection

At 2 h, 2 days, and 1 week after NMDA injection, the rats were euthanized, and the hippocampus was dissected out for Western blot analysis. The tissue samples were weighted and kept at −70 °C until analysis.

For the immunohistochemistry, the rats were transcardially perfused with 0.05 M phosphate-buffered saline (PBS; pH 7.4) and then 4% paraformaldehyde solution (PFA). The fixed brain samples were kept in 30% sucrose over 2 days, and sectioned in the coronal direction (40 μm) on a freezing microtome at −26 ± 2 ℃.

### 2.5. Western Blot Analysis

Brain tissues were homogenized in RIPA buffer, including 1 mM phenyl methyl sulfonyl fluoride (PMSF) and 1× protease/phosphatase inhibitor cocktail (Cell Signaling Technology, Danvers, MA, USA). The protein concentration was assessed using Bradford reagent (Sigma-Aldrich, St. Louis, MO, USA). Equal amounts of proteins (50 μg) were separated on SDS-PAGE and were transferred on nitrocellulose membranes (Amersham Biosciences, Uppsala, Sweden). The membranes were blocked with 5% skim milk and incubated overnight with rabbit-cleaved CASP3 or mouse GFAP antibodies (Cell Signaling Technology) at 4 °C. Horseradish peroxidase-conjugated anti-rabbit or anti-mouse IgG was used as a secondary antibody. The protein detection was performed using the enhanced chemiluminescence (ECL) detection solution (Bio-Rad, Hercules, CA, USA). The detected bands were quantified using ImageJ (NIH, Bethesda, Rockville, MD, USA).

### 2.6. Nissl Staining

The brain sections involved with the hippocampus were selected. The sections were mounted on gelatin-coated slides and were dried in air. For staining, cresyl violet (Sigma-Aldrich) was dissolved in distilled water with 0.25% acetic acid. The slide-mounted brain sections were soaked into 0.1% cresyl violet solution for 2 min and were covered with coverslips.

### 2.7. Immunohistochemistry

For immunohistochemistry, the floating sections were treated with 1% dihydrogen dioxide, and blocked with 10% normal goat or horse serum (Vector Laboratories, Burlingame, CA, USA) including 0.1% BSA (bovine serum albumin). The sections were probed with mouse neuronal nuclei (NeuN) (Millipore, Burlington, MA, USA), rabbit-cleaved CASP3, or mouse GFAP antibodies (Cell Signaling Technology) overnight at 4 °C. The sections were incubated with the biotinylated horse anti-mouse IgG or goat anti-rabbit IgG for 1 h, and then with the Vector Elite ABC Kit (Vector Laboratories) for 30 min. After DAB staining (Vector laboratories), the sections were mounted onto gelatin-coated slides and covered with coverslips using permount (Fisher Scientific, Pittsburgh, PA, United States). Immunohistochemical images were visualized under a light microscope (Olympus BX43, Shinjuku, Tokyo, Japan). On the immunostaining of CASP3 and GFAP, the staining density in the sections was quantitatively measured using ImageJ (NIH). On the immunostaining of NeuN, the number of NeuN-positive cells was counted from each section, and cell counts were presented as the mean number of cells per unit area (mm^2^).

### 2.8. Terminal, Deoxynucleotidyl Transferase-Mediated, dUTP–Biotin, Nick End Labeling (TUNEL) Staining

Terminal, deoxynucleotidyl transferase-mediated, dUTP–biotin, nick end labeling (TUNEL) staining was performed in the hippocampus using the In Situ Cell Death Detection Kit (Roche Applied Science, Penzberg, Germany) according to the manufacturer’s protocol. Brain sections were permeabilized with 0.5% Triton X-100 and then incubated in the TUNEL reaction mixture containing terminal deoxynucleotidyl transferase and nucleotide mixture for 1 h at 37 °C. The staining was visualized using Converter-POD with DAB (Vector Laboratories). TUNEL-positive cells were counted from each section, and cell counts were presented as the mean number of cells per unit area (mm^2^).

### 2.9. Statistical Analysis

The results are presented as the means ± standard deviation (SD). Differences in the results were analyzed using Kruskal–Wallis test with SPSS statistics 25 (IBM, Chicago, IL, USA). In the conductivity analysis of MREPT, the *p*-values were adjusted according to the false discovery rate (FDR) using the Benjamini–Hochberg algorithm for multiple comparison correction. The *p*-values of <0.05 were considered statistically significant.

## 3. Results

### 3.1. Seizure Severity after NMDA Treatment

**Figure 1:**
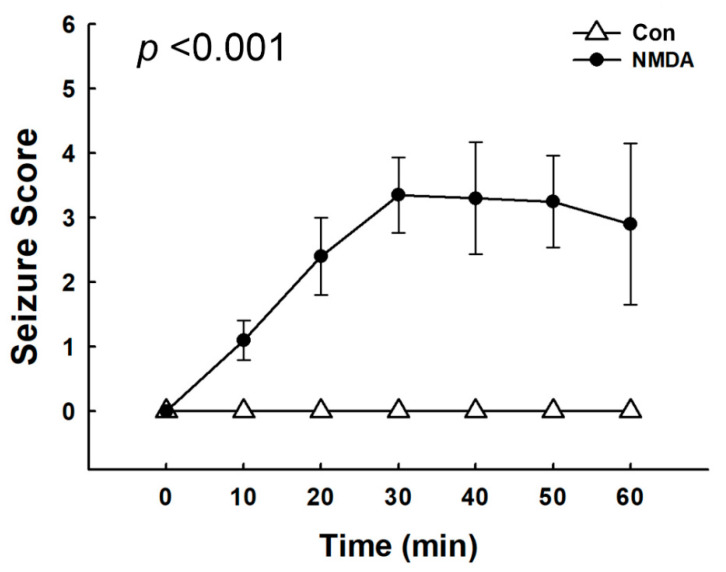
Assessment of the seizure severity in N-methyl-D-aspartate (NMDA)-treated rats. Seizures were immediately triggered in rats following NMDA treatment (75 mg/kg). The scores of seizure severity were assessed every 10 min based on a modified Racine’s scale. The severity of seizures was increased time-dependently. Seizure score: 0 = normal; 1 = motionless; 2 = tail stiffness; 3 = lordotic posture and whip-like tail; 4 = forelimb clonus with rearing and falling, body twist; and 5 = generalized tonic–clonic activity.

The severity of seizures was scored based on a modified Racine’s scale every 10 min for 1 h. The seizure scores gradually increased after NMDA treatment (Figure 1). After NMDA treatment, the rats were motionless at 10 min, and then showed stiff tails at 20 min. The highest seizure scores (scores 3 to 5) were shown at approximately 30 min after NMDA treatment. Twisting of the whole body, clonus with a rearing posture, including continuous whip-like tail movement, or generalized tonic–clonic seizures were observed. Seizures were prominently sustained for 1 h after NMDA treatment, and then entirely abolished within 90 min in all of the rats.

### 3.2. Change of Conductivity in the Brain of NMDA-Treated Rats

**Figure 2:**
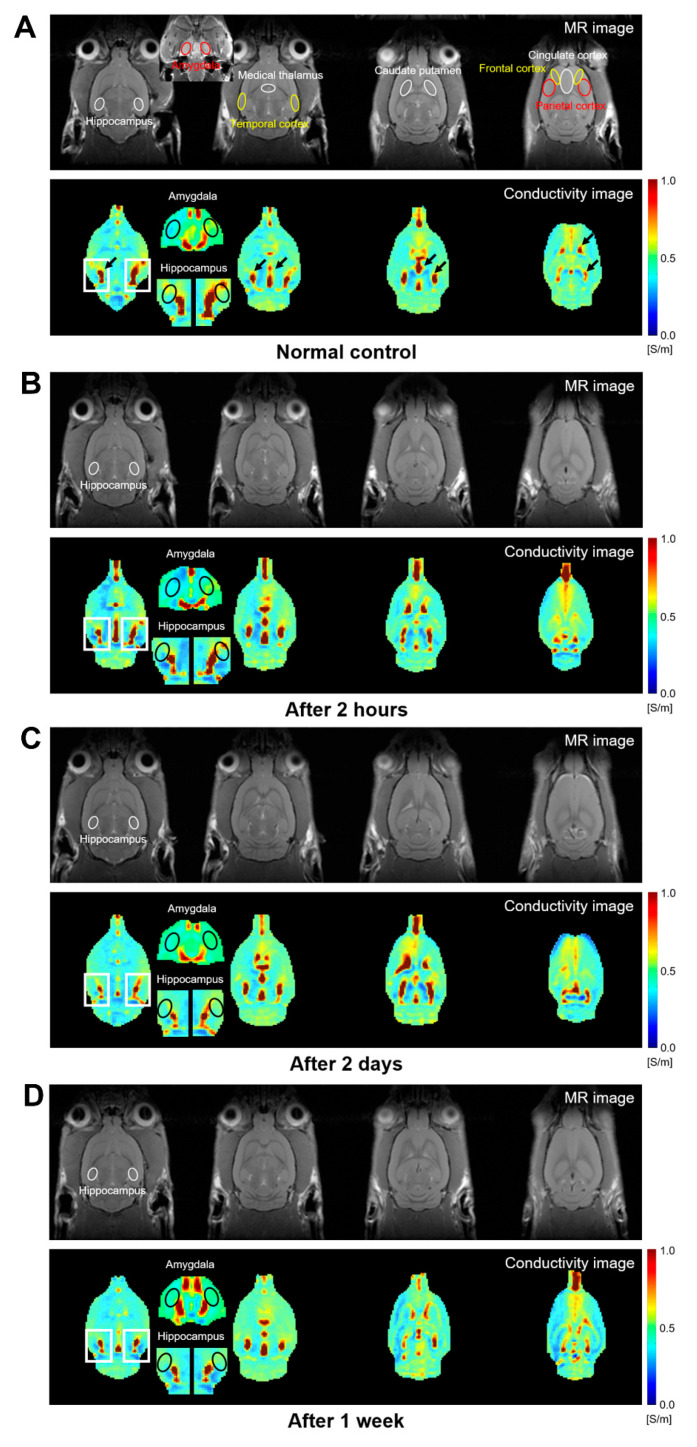
Magnetic resonance (MR) and corresponding conductivity images of rat brains using 9.4 T magnetic resonance imaging (MRI). The imaging results are from four groups representing (**A**) normal control, (**B**) 2 h, (**C**) 2 days, and (**D**) 1 week after the NMDA treatment, respectively. The hippocampal region was enlarged and marked the regions of interest (ROIs) in the conductivity image. The black arrows indicate the highest conductivity in the CSF (cerebrospinal fluid).

Using ultra-high-field (9.4 T) MRI, the conductivity in the rat brains after NMDA treatment was reconstructed through the MRCI toolbox. The proton density-weighted MR images indicate the anatomical structures and MR tissue contrast of the rat brains (Figure 2A–D, upper part of each). The pseudo color images on electrical conductivity show the conductivity contrasts (Figure 2A–D, lower part of each). The highest electrical conductivity was found in the CSF (marked as black arrows in Figure 2A). Overall, we found that the conductivity contrast was different between the control and NMDA-treated rats in various brain regions.

In particular, we selected several brain regions where the difference of the conductivity contrast was potently shown between the control and NMDA-treated rats, or those that were intensively studied for seizures [6,12,13,14], and quantified the conductivity in ROIs for the selected brain regions (Table 1). Among the ROIs, we found a significant difference of conductivity in the hippocampus (*p* = 0.011). In the hippocampus, the conductivity decreased at 2 h, 2 days, and 1 week after the NMDA treatment compared to the control. The conductivity decreased the most at 2 h (0.433 ± 0.016 S/m), and thereafter slightly increased (0.444 ± 0.013 at 2 days and 0.442 ± 0.014 S/m at 1 week). However, the significant difference disappeared after multiple-comparison correction by the Benjamini–Hochberg algorithm.

### 3.3. Morphological Changes and Neuronal Loss in the Hippocampus of NMDA-Treated Rats

**Figure 3:**
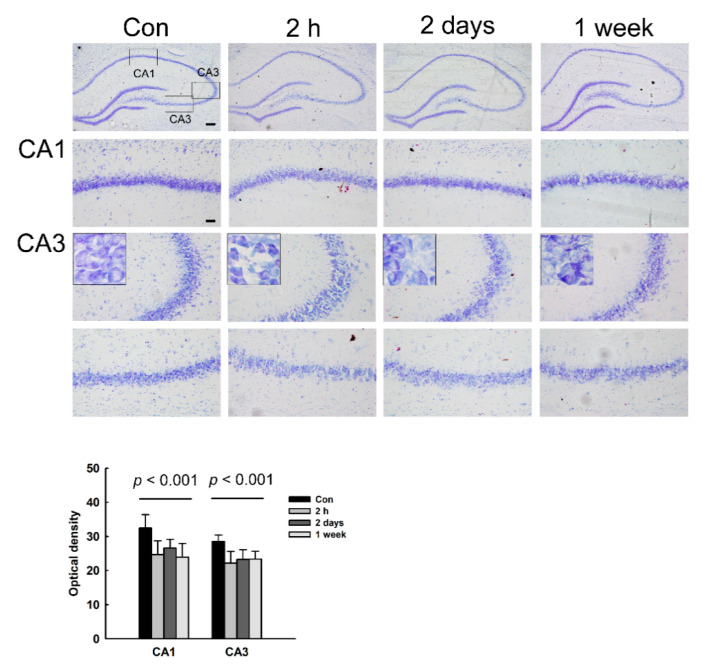
Neuronal cell death in the hippocampus of NMDA-treated rats. Morphological changes in the hippocampus after NMDA treatment were assessed using Nissl staining. The histogram reveals the optical density of Nissl-stained cells as the mean ± standard error of the mean (SEM). Scale bars = 200 (low magnification) or 50 µm (high magnification).

The morphological changes in NMDA-treated rats were observed in the brain regions quantified by the conductivity using Nissl staining. We found significant morphological changes and neuronal loss in the hippocampus of NMDA-treated rats (Figure 3). At 2 h after NMDA treatment, neuronal loss was shown in the CA1 and CA3 regions of the hippocampus. In addition, shrunken cell bodies and condensed cytoplasm were revealed, particularly in the neurons of the CA3 region (see the highest magnification). At 2 days and 1 week after NMDA treatment, the marked cell shrinkage and cytoplasmic condensation were not observed; however, consistent neuronal cell loss were shown in the CA1 and CA3 regions.

In other brain regions, we did not observe a significant morphological change or neuronal loss (data not shown).

### 3.4. Decrease of NeuN Immunoreactivity (IR) in the Hippocampus of NMDA-Treated Rats

**Figure 4:**
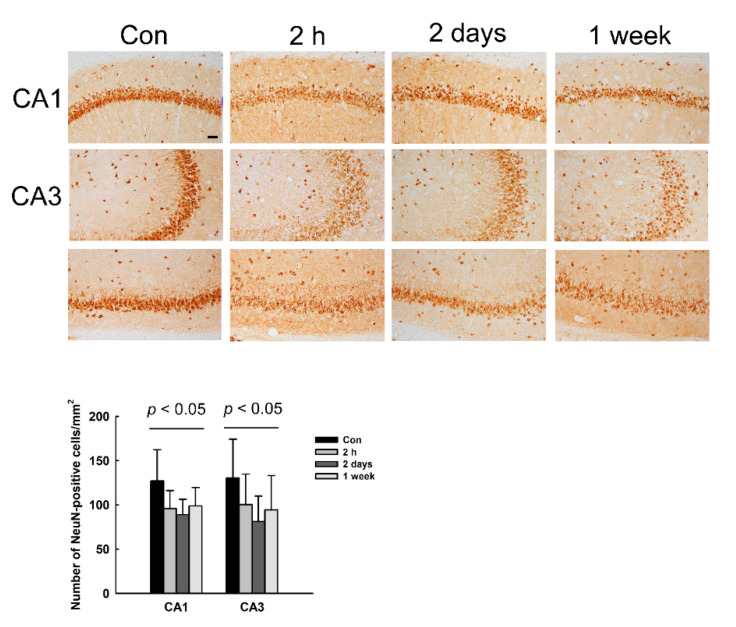
Immunoreactivity (IR) of neuronal nuclei (NeuN) in the hippocampus of NMDA-treated rats. NeuN-IR is observed in the nuclei of neurons. NeuN-positive cells were counted in the hippocampal CA1 and CA3 regions. The histogram presents the number of NeuN-positive cells per mm^2^ as the mean ± SD. Scale bars indicate 50 µm.

In order to confirm the neuronal loss in the hippocampus of NMDA-treated rats shown by the Nissl staining, we examined immunoreactivity (IR) on a neuronal marker NeuN. As shown in Figure 4, NeuN IR was observed in the nuclei of neurons. The number of NeuN-positive cells decreased in the CA1 and CA3 regions of the hippocampus at all time points after NMDA treatment (2 h, 2 days, and 1 week), compared to control. This result indicates that NMDA induces neuronal loss in the hippocampus, which is sustained from 2 h to 1 week after NMDA treatment.

### 3.5. Induction of Apoptotic Cell Death in the Hippocampus of NMDA-Treated Rats

**Figure 5:**
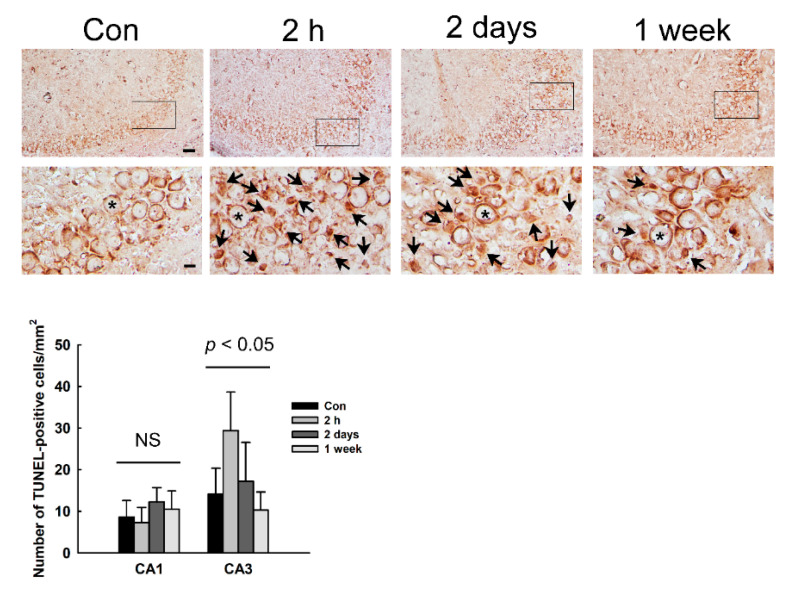
Apoptotic cell death in the hippocampus of NMDA. Apoptotic cell death was detected using terminal, deoxynucleotidyl transferase-mediated, dUTP–biotin, nick end labeling (TUNEL) staining. Arrows and asterisks indicate TUNEL-positive and -negative cells, respectively. Results are expressed as mean number of TUNEL-positive cells ± SD. Scale bars = 50 (low magnification) or 10 µm (high magnification). NS: not significant.

Positive TUNEL staining indicates apoptotic DNA fragmentation. In order to detect the apoptotic cells, we performed TUNEL staining in the hippocampus. We found that the number of TUNEL-positive cells increased in the CA3 region of the hippocampus at 2 h and 2 days after NMDA treatment (Figure 5). In particular, TUNEL-positive cells were most abundant at 2 h after NMDA treatment. However, the number of TUNEL-positive cells in the CA1 region was not different between control and NMDA-treated rats.

**Figure 6:**
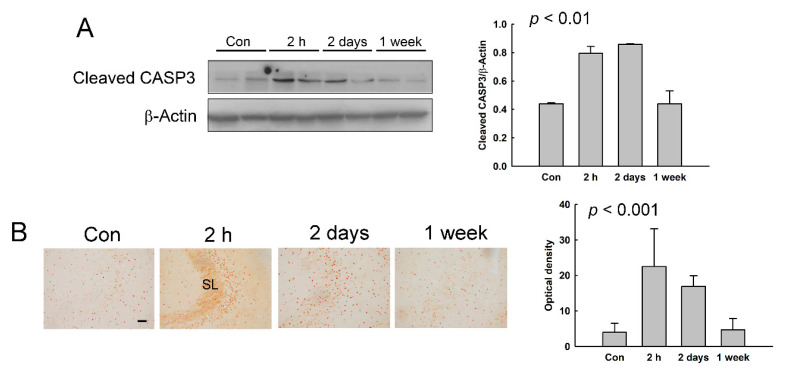
The expressions of cleaved caspase 3 (CASP3) in the hippocampus of NMDA-treated rats. (**A**) The expressions of cleaved CASP3 in the hippocampus were assessed using western blotting. The histograms represent the protein expression levels divided by the expression level of β-actin as the mean ± SD. β-Actin was used as an internal control. (**B**) Cleaved CASP3-immunoreactivity (IR) was examined in the hippocampus. The optical density of cleaved CASP3-IR is shown as the mean ± SD. Scale bars = 50 µm. SL, stratum lucidum.

We also investigated the expression of cleaved CASP3, an apoptotic executor, in the hippocampus of NMDA-treated rats. The protein expression of cleaved CASP3 was increased in the hippocampus at 2 h and 2 days after NMDA treatment, with the peak expression at 2 h (Figure 6A). Cleaved CASP3 IR was detected in the CA3 region of the hippocampus of NMDA-treated rats (Figure 6B). Cleaved CASP3 IR was up-regulated at 2 h and 2 days after NMDA treatment in the CA3, especially in the stratum lucidum of the CA3 region, with the peak IR at 2 h. These results indicates that seizure-induced apoptotic cell death occurs for 2 h to 2 days after NMDA treatment (profoundly at 2 h) in the hippocampus.

### 3.6. Increase of Reactive Astrocytes in the Hippocampus of NMDA-Treated Rats

**Figure 7:**
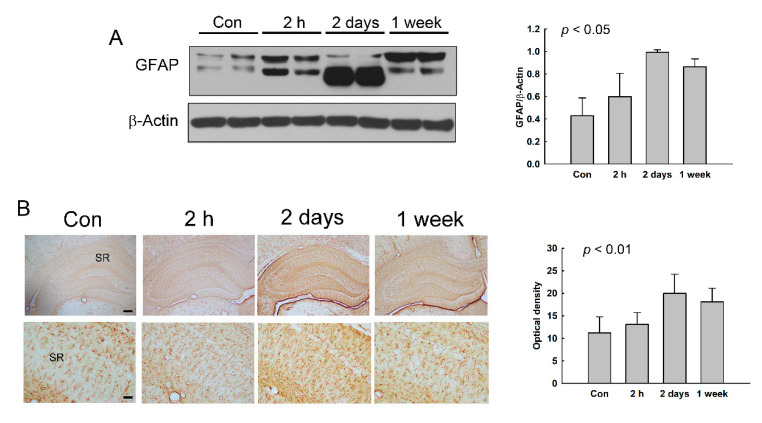
The expression of glial fibrillary acidic protein (GFAP) in the hippocampus of NMDA-treated rats. (**A**) The expression of GFAP in the hippocampus was assessed using Western blotting. The histograms represent the protein expression levels divided by the expression level of β-actin as the mean ± SD. β-Actin was used as an internal control. (**B**) The GFAP immunoreactivity (IR) was examined in the hippocampus. The optical density of GFAP IR is shown as the mean ± SD. Scale bars indicate 200 (low magnification) or 50 µm (high magnification). SR: stratum radiatum.

We speculated that the slight increase of conductivity at 2 days and 1 week after NMDA treatment may be caused by astrogliosis after the brain damage. Thus, we examined the expression of GFAP, which is a hallmark of reactive astrocytes [25,26], in the hippocampus of NMDA-treated rats. As shown in Figure 7A, the protein expression of GFAP was increased in the hippocampus at 2 days and 1 week after NMDA treatment. GFAP-IR was also elevated in the hippocampus at 2 days and 1 week (Figure 7B). In particular, the elevation was potent in the stratum radiatum of the CA1 region, like previously shown in seizure rodents [27,28]. These results indicate that the reactive astrocytes were increased in the hippocampus from 2 days after seizure.

## 4. Discussion

In this study, we investigated the possibility of MREPT as an applicable technique to detect the alterations of morphology and biological molecules in seizure-induced damaged brain using NMDA-treated rats. Through ultra-high-field (9.4 T) MRI scans in the brain of NMDA-treated rats, we found that conductivity was reduced in the hippocampus after seizure. We also observed that NMDA treatment induced neuronal cell death in the hippocampus, along with increasing TUNEL-positive cells and the expression levels of cleaved CASP3.

MREPT has an advantage to imaging electrical tissue properties related to the ionic concentration and mobility of intra- and extracellular space at a high frequency. The change of high-frequency conductivity using MREPT has been revealed to represent the alteration of tissue composition, such as the vasculature, body fluid, or protein content in brain tumors [29]. A study on an ischemic stroke patient using MREPT suggested that an elevation of conductivity in the infarction area two months after the infarction was caused by viable brain parenchyma [30]. In addition, the high-frequency conductivity of MREPT was shown to gradually decrease as time passed in the canine brain after euthanasia, together with the gradual destruction of the brain tissue [19]. In our study, the ultra-high-field (9.4 T) MRI scanner results showed that conductivity was reduced in the hippocampus after seizure. Given the previous in vivo studies using MREPT [19,29,30], this decrease of conductivity after a seizure in the hippocampus may be associated with the seizure-induced changes in the morphology or biochemical molecules of the hippocampus. Interestingly, the hippocampus is a brain region that has been intensively studied in seizure-induced neuronal cell death [4,6,9,12]. Our results also show that neuronal cell death was induced in the hippocampus of NMDA-treated seizure rats. At all time points after NMDA treatment, neuronal cell loss was observed in the hippocampus via Nissl staining and a decrease of NeuN-positive cells. In particular, at 2 h after NMDA treatment, Nissl staining showed the morphological features of apoptotic cell death, such as shrunken cell bodies and cytoplasmic condensation, predominantly in the CA3 region of the hippocampus. TUNEL-positive cells and the expression of cleaved CASP3, an apoptosis executor, were also most potently increased in the CA3 region of the hippocampus at 2 h after NMDA treatment. However, at 1 week after NMDA treatment, despite neuronal cell loss, TUNEL-positive cells were relatively rare compared to at 2 h and 2 days, and the expression of cleaved CASP3 was not detected. Thus, alterations in the biochemical molecules related to apoptotic cell death may be predominantly induced in the hippocampus at 2 h after NMDA-induced seizure. From there, up to 1 week, the neuronal loss may be sustained in the hippocampus without an obvious recovery. Thus, the decreased conductivity of MREPT at all time points may represent neuronal loss by seizures.

However, intriguingly, the conductivity was most potently decreased at 2 h after NMDA treatment (0.433 S/m), and thereafter slightly increased (0.444 S/m at 2 days and 0.442 S/m at 1 week). This change of the conductivity might be also caused by the morphological and biochemical alterations during and after neuronal cell death. As mentioned above, the most potent decrease of the conductivity at 2 h after NMDA treatment might be associated with the induction of apoptotic cell death. In addition, we expect that the slight increase of conductivity might be related to astrogliosis. Astrocytes are the most numerous cells in the brain, and they play a role in the homeostasis of the extracellular glutamate level [31], ion/pH [32], and brain–blood barrier integrity [33]. Recent studies have revealed that astrogliosis, a process where surviving astrocytes after a brain injury become hypertrophic and proliferate in the affected region [34], was induced in the hippocampus after brain damage from a seizure [27,28]. Reactive astrocytes play two contrary roles after brain damage: inhibiting dendritic and axonal remodeling in neuronal circuits [35,36], and also releasing many growth factors and trophic factors, promoting neuronal survival, synaptogenesis, and neurogenesis after brain damage [35,37]. In particular, astrogliosis after a seizure was induced predominantly in the stratum radiatum of the CA1 region of the hippocampus, showing increased expression of GFAP [27,28]. In our study, we also found that the expression of GFAP was increased in the hippocampus, including the stratum radiatum of the CA1 region at 2 days and 1 week after seizure. The slight increase of conductivity might be caused by the elevated reactive astrocytes after seizure, which altered the synaptic levels of neurotransmitters and ions. Our results for the amygdala also support this. In the amygdala, conductivity was increased at 2 days and 1 week after NMDA treatment, with only a slight decrease at 2 h, although a statistical significance was not detected. NMDA did not induce significant neuronal cell death in the amygdala, although a slight decrease in the optical density of Nissl staining was shown at 2 h after NMDA treatment (Supplementary Figure S1). However, the GFAP expression in the amygdala was significantly increased at 2 days and 1 week after NMDA treatment (Supplementary Figure S2). Although predominant cell death was not induced, the increase of reactive astrocytes after the slight neuronal cell death by NMDA treatment might result in the elevated conductivity in the amygdala.

The advantage of the conductivity imaging provides new contrast information compared to the existing imaging method. The contrast mechanism of electrical conductivity originates from the changes of concentration and mobility of ions in the intracellular and extracellular space [19,38]. The other factor is the cellular conditions, such as cell density and anisotropy [19,38]. Given our histological findings and the conductivity images, the decreased conductivity of the hippocampus in NMDA treatment groups may be related to neuronal loss or cell destruction, which may affect the concentration and mobility of ions. The electrical conductivity changes are closely related to the physiological and pathological conditions of tissues and organs [19,38]. However, since the conductivity imaging method can be implemented by using an existing MRI system, there are the resolution and sensitivity issues of MRI images. Moreover, even if the conductivity of brain tissue can provide information on the microscopic changes at a macroscopic level, there are still limitations in explaining the microscopic changes. Further studies and analysis are needed to confirm the effect of each microscopical change on brain conductivity, such as apoptosis and astrogliosis. We expect that a further study using the high- and low-frequency electrical properties that can simultaneously measure high and low frequencies [39] could distinguish the changes of conductivity caused by cell loss and astrogliosis.

## 5. Conclusions

In conclusion, our results showed that seizures decreased high-frequency conductivity, through MREPT in the hippocampus. The seizure-induced decrease of conductivity might be caused by neuronal loss in the hippocampus. Interestingly, conductivity was decreased the most at 2 h after NMDA treatment, and thereafter slightly increased. This change of the conductivity might represent alterations in the apoptosis- or astrogliosis-related biochemicals during and after brain damage following seizures. Taken together, these results indicate that MREPT may be an applicable technique to non-invasively identify the changes of the morphology and biological molecules in the damaged brain after a seizure.

There were some limitations to our study. In the analysis of the conductivity of MREPT, the difference of the conductivity among control and NMDA-treated rats did not remain after multiple comparison corrections. In addition, our study was performed using a relatively small sample size. Moreover, because the results are not derived from the same rats over time, it would be speculative to compare between different time points. Further studies are needed with a larger sample size and a stepping protocol with partial rat sacrifice (30 scanned at baseline and 10 sacrificed, 20 scanned at 2 days and another 10 sacrificed, etc.).

## Figures and Tables

**Table 1:** High-frequency conductivity in the brain regions of NMDA-treated rats.

Region	Conductivity (S/m)				*p*	FDR *p*
	Control	2 h	2 Days	1 Week		
Amygdala	0.434 ± 0.025	0.418 ± 0.011	0.446 ± 0.025	0.480 ± 0.013	0.058	0.091
Medial thalamus	0.399 ± 0.016	0.392 ± 0.006	0.403 ± 0.021	0.423 ± 0.010	0.131	0.234
Hippocampus	0.501 ± 0.038	0.433 ± 0.016	0.444 ± 0.013	0.442 ± 0.014	0.011 *	0.261
Temporal cortex	0.441 ± 0.003	0.433 ± 0.004	0.458 ± 0.015	0.466 ± 0.019	0.126	0.261
Frontal cortex	0.459 ± 0.013	0.455 ± 0.010	0.433 ± 0.026	0.447 ± 0.015	0.516	0.510
Parietal cortex	0.434 ± 0.026	0.429 ± 0.005	0.415 ± 0.009	0.417 ± 0.023	0.553	0.510
Cingulate cortex	0.538 ± 0.003	0.544 ± 0.050	0.536 ± 0.031	0.575 ± 0.028	0.383	0.553
Caudate putamen	0.420 ± 0.021	0.433 ± 0.014	0.443 ± 0.040	0.465 ± 0.018	0.357	0.553

* *p* < 0.05 compared to the control group. S: Siemens; m: meter; FDR: false discovery rate.

## Supplementary Materials

The following are available online at https://www.mdpi.com/2075-4418/11/3/569/s1, Figure S1: Neuronal cell death in the amygdala of NMDA-treated rats, Figure S2: The expression of glial fibrillary acidic protein (GFAP) in the amygdala of NMDA-treated rats.

## References

[1] Stafstrom C.E., Carmant L. (2015). Seizures and epilepsy: An overview for neuroscientists. Cold Spring Harb. Perspect. Med..

[2] Chapman A.G. (2000). Glutamate and epilepsy. J. Nutr..

[3] Walker M.C. (2018). Pathophysiology of status epilepticus. Neurosci. Lett..

[4] During M.J., Spencer D.D. (1993). Extracellular hippocampal glutamate and spontaneous seizure in the conscious human brain. Lancet.

[5] Furness A.M., Pal R., Michealis E.K., Lunte C.E., Lunte S.M. (2019). Neurochemical investigation of multiple locally induced seizures using microdialysis sampling: Epilepsy effects on glutamate release. Brain Res..

[6] Barker-Haliski M., White H.S. (2015). Glutamatergic Mechanisms Associated with Seizures and Epilepsy. Cold Spring Harb. Perspect. Med..

[7] Sharma S., Puttachary S., Thippeswamy A., Kanthasamy A.G., Thippeswamy T. (2018). Status epilepticus: Behavioral and electroencephalography seizure correlates in kainate experimental models. Front. Neurol..

[8] Lau A., Tymianski M. (2010). Glutamate receptors, neurotoxicity and neurodegeneration. Pflug. Arch. Eur. J. Physiol..

[9] Park H.J., Kim H.J., Park H.J., Ra J., Zheng L.T., Yim S.V., Chung J.H. (2008). Protective effect of topiramate on kainic acid-induced cell death in mice hippocampus. Epilepsia.

[10] Cao J., Viholainen J.I., Dart C., Warwick H.K., Leyland M.L., Courtney M.J. (2005). The PSD95-nNOS interface: A target for inhibition of excitotoxic p38 stress-activated protein kinase activation and cell death. J. Cell Biol..

[11] Wang Y., Dong X.X., Cao Y., Liang Z.Q., Han R., Wu J.C., Gu Z.L., Qin Z.H. (2009). p53 induction contributes to excitotoxic neuronal death in rat striatum through apoptotic and autophagic mechanisms. Eur. J. Neurosci..

[12] Cianfoni A., Caulo M., Cerase A., Della Marca G., Falcone C., Di Lella G.M., Gaudino S., Edwards J., Colosimo C. (2013). Seizure-induced brain lesions: A wide spectrum of variably reversible MRI abnormalities. Eur. J. Radiol..

[13] Kim H., Choi Y., Joung H.-Y., Choi Y.S., Kim H.J., Joo Y., Oh J.-H., Hann H.J., Cho Z.-H., Lee H.W. (2017). Structural and functional alterations at pre-epileptic stage are closely associated with epileptogenesis in pilocarpine-induced epilepsy model. Exp. Neurobiol..

[14] Hübers A., Thoma K., Schocke M., Fauser S., Ludolph A.C., Kassubek J., Pinkhardt E.H. (2018). Acute DWI reductions in patients after single epileptic seizures-more common than assumed. Front. Neurol..

[15] Maganti R.K., Rutecki P. (2013). EEG and epilepsy monitoring. Continuum.

[16] Hannan S., Faulkner M., Aristovich K., Avery J., Holder D. (2018). Frequency-dependent characterisation of impedance changes during epileptiform activity in a rat model of epilepsy. Physiol. Meas..

[17] Kwon O.I., Jeong W.C., Sajib S.Z., Kim H.J., Woo E.J., Oh T.I. (2014). Reconstruction of dual-frequency conductivity by optimization of phase map in MREIT and MREPT. Biomed. Eng. Online.

[18] Kim D.H., Chauhan M., Kim M.O., Jeong W.C., Kim H.J., Sersa I., Kwon O.I., Woo E.J. (2015). Frequency-dependent conductivity contrast for tissue characterization using a dual-frequency range conductivity mapping magnetic resonance method. IEEE Trans. Med. Imaging.

[19] Sajib S.Z.K., Kwon O.I., Kim H.J., Woo E.J. (2018). Electrodeless conductivity tensor imaging (CTI) using MRI: Basic theory and animal experiments. Biomed. Eng. Lett..

[20] Lüttjohann A., Fabene P.F., van Luijtelaar G. (2009). A revised Racine’s scale for PTZ-induced seizures in rats. Physiol. Behav..

[21] Ihara Y., Tomonoh Y., Deshimaru M., Zhang B., Uchida T., Ishii A., Hirose S. (2016). Retigabine, a Kv7. 2/Kv7. 3-channel opener, attenuates drug-induced seizures in knock-in mice harboring Kcnq2 mutations. PLoS ONE.

[22] Bioucas-Dias J.M., Valadão G. (2007). Phase unwrapping via graph cuts. IEEE Trans. Image Process. Publ. IEEE Signal Process. Soc..

[23] Sajib S.Z., Katoch N., Kim H.J., Kwon O.I., Woo E.J. (2017). Software toolbox for low-frequency conductivity and current density imaging using MRI. IEEE Trans. Biomed. Eng..

[24] Paxinos G., Watson C. (2006). The Rat Brain in Stereotaxic Coordinates: Hard Cover Edition.

[25] Meini A., Sticozzi C., Massai L., Palmi M. (2008). A nitric oxide/Ca^2+^/calmodulin/ERK1/2 mitogen-activated protein kinase pathway is involved in the mitogenic effect of IL-1β in human astrocytoma cells. Br. J. Pharmacol..

[26] Sticozzi C., Belmonte G., Meini A., Carbotti P., Grasso G., Palmi M. (2013). IL-1β induces GFAP expression in vitro and in vivo and protects neurons from traumatic injury-associated apoptosis in rat brain striatum via NFκB/Ca^2+^-calmodulin/ERK mitogen-activated protein kinase signaling pathway. Neuroscience.

[27] Kim D.S., Kim J.E., Kwak S.E., Choi K.C., Kim D.W., Kwon O.S., Choi S.Y., Kang T.C. (2008). Spatiotemporal characteristics of astroglial death in the rat hippocampo-entorhinal complex following pilocarpine-induced status epilepticus. J. Comp. Neurol..

[28] Kim J.-E., Kang T.-C. (2018). Nucleocytoplasmic p27Kip1 export is required for ERK1/2-mediated reactive astroglial proliferation following status epilepticus. Front. Cell. Neurosci..

[29] Voigt T., Vaterlein O., Stehning C., Katscher U., Fiehler J. In vivo glioma characterization using MR conductivity imaging. Proceedings of the 19th Scientific Meeting of the International Society of Magnetic Resonance in Medicine (ISMRM’11).

[30] van Lier A., Kolk A., Brundel M., Hendriske J., Luijten J., Lagendijk J., van den Berg C. Electrical conductivity in ischemic stroke at 7.0 Tesla: A case study. Proceedings of the 20th Scientific Meeting of the International Society of Magnetic Resonance in Medicine (ISMRM’12).

[31] Anderson C.M., Swanson R.A. (2000). Astrocyte glutamate transport: Review of properties, regulation, and physiological functions. Glia.

[32] Simard M., Nedergaard M. (2004). The neurobiology of glia in the context of water and ion homeostasis. Neuroscience.

[33] Takano T., Tian G.-F., Peng W., Lou N., Libionka W., Han X., Nedergaard M. (2006). Astrocyte-mediated control of cerebral blood flow. Nat. Neurosci..

[34] Ridet J., Privat A., Malhotra S., Gage F. (1997). Reactive astrocytes: Cellular and molecular cues to biological function. Trends Neurosci..

[35] Horner P.J., Gage F.H. (2000). Regenerating the damaged central nervous system. Nature.

[36] Rossi D.J., Brady J.D., Mohr C. (2007). Astrocyte metabolism and signaling during brain ischemia. Nat. Neurosci..

[37] Panickar K.S., Norenberg M.D. (2005). Astrocytes in cerebral ischemic injury: Morphological and general considerations. Glia.

[38] Seo J.K., Woo E.J. (2014). Electrical tissue property imaging at low frequency using MREIT. IEEE Trans. Biomed. Eng..

[39] Jahng G.H., Lee M.B., Kim H.J., Woo E.J., Kwon O.I. (2021). Low-frequency dominant electrical conductivity imaging of in vivo human brain using high-frequency conductivity at Larmor-frequency and spherical mean diffusivity without external injection current. NeuroImage.

