# Supplementary material for: Identification of Brain Damage after Seizures Using an MR-Based Electrical Conductivity Imaging Method
Source: Diagnostics (Basel). 2021 Mar 22;11(3):569. doi: 10.3390/diagnostics11030569 (PMC8004663; doi:10.3390/diagnostics11030569)
Supplement: Supplementary file 1 [file diagnostics-11-00569-s001.pdf]

Supplementary Figure S1

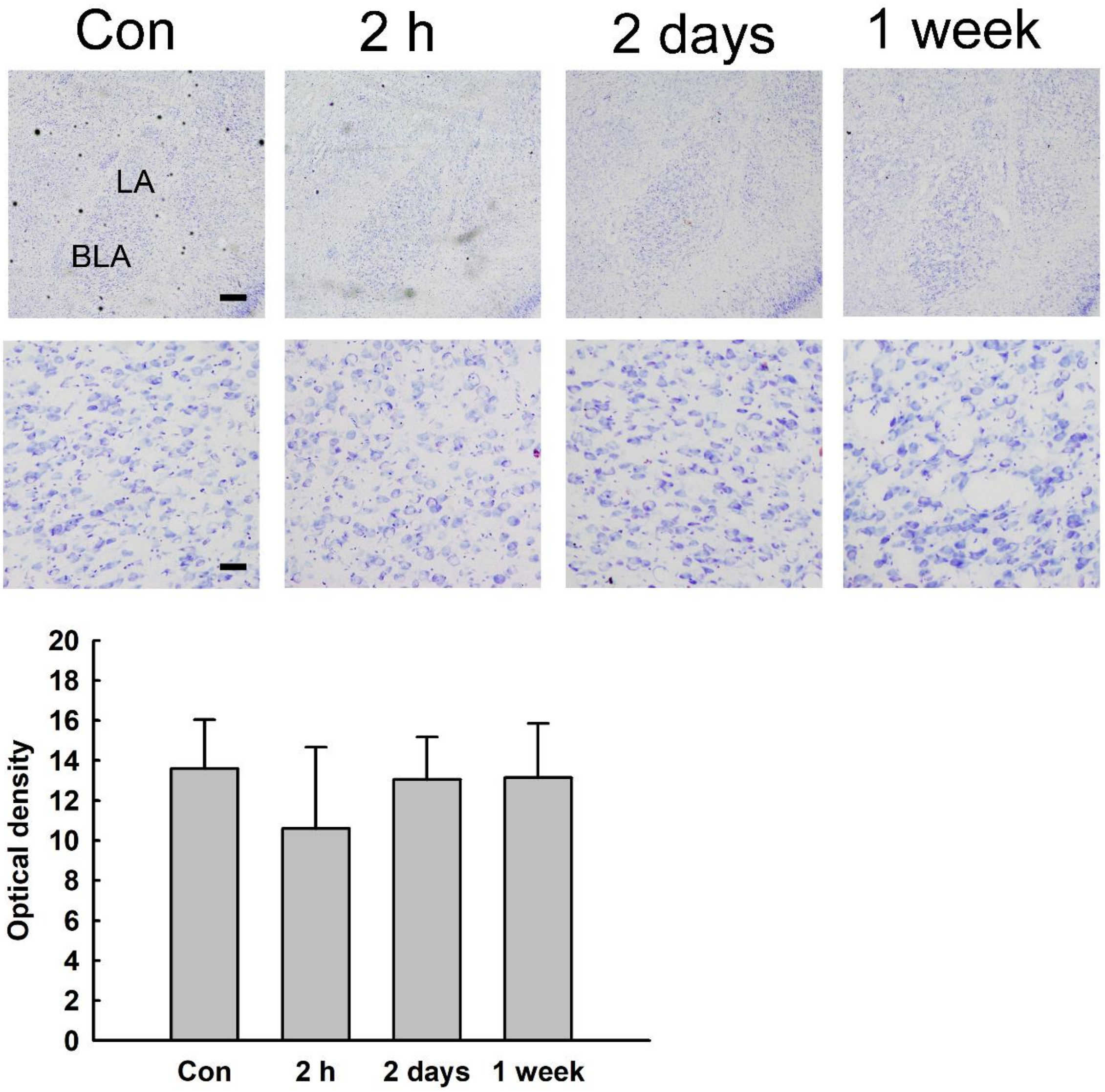

Neuronal cell death in the amygdala of NMDA-treated rats. Morphological changes in the amygdala after NMDA treatment were assessed using Nissl staining. High magnification shows Nissl-stained cells in the basolateral amygdala. The histogram reveals the optical density of Nissl-stained cells as the mean ± standard error of the mean (SEM). Scale bars indicate 200 (low magnification) or 50 μm (high magnification). *$p < 0.05$ compared to the control group. LA, lateral amygdala; BLA, basolateral amygdala.

Supplementary Figure S2

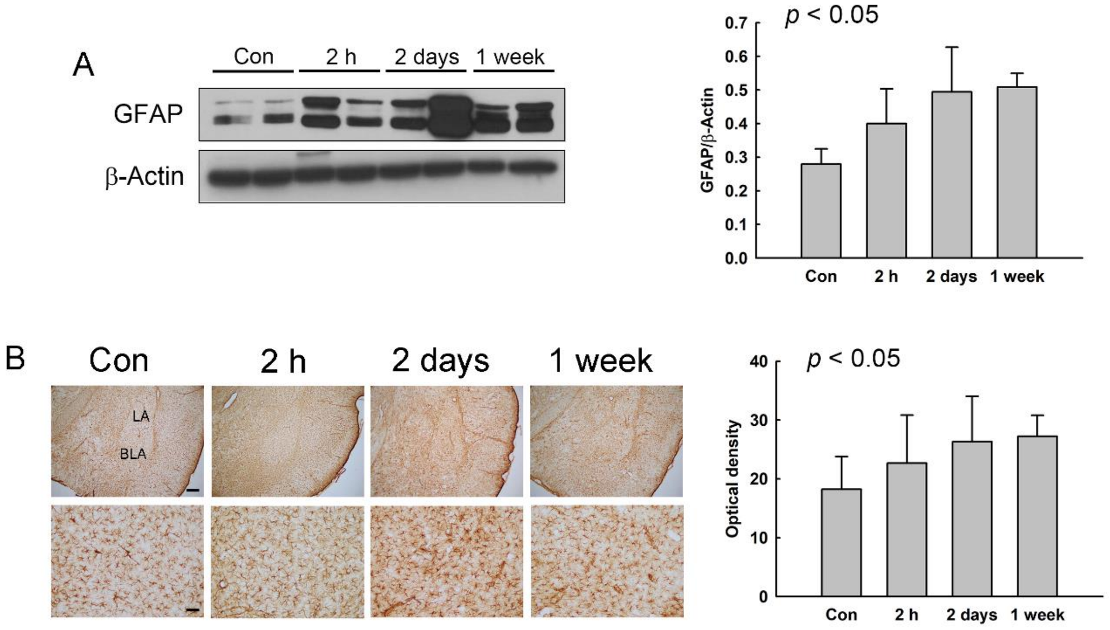

The expression of glial fibrillary acidic protein (GFAP) in the amygdala of NMDA-treated rats. (a) The expression of GFAP in the amygdala was assessed using western blotting. The histograms represent the protein expression levels divided by the expression level of β-actin as the mean ± SEM. β-Actin was used as an internal control. (b) The GFAP-immunoreactivity (IR) was examined in the amygdala. High magnification shows the GFAP-IR in the basolateral amygdala. The optical density of GFAP-IR is shown as the mean ± SEM. Scale bars indicate 200 (low magnification) or 50 μm (high magnification). *p < 0.05 compared to the control group. LA, lateral amygdala; BLA, basolateral amygdala.
